# Association of potentially inappropriate medications and need for long-term care among older adults: a matched cohort study

**DOI:** 10.1186/s12877-022-03681-5

**Published:** 2022-12-16

**Authors:** Suhyun Jang, Hee-Jin Kang, Yeji Kim, Sunmee Jang

**Affiliations:** 1 College of Pharmacy and Gachon Institute of Pharmaceutical Sciences, Gachon University, 191 Hambakmoe‑Ro, Yeonsu‑Gu, Incheon, 21936 Republic of Korea; 2 Clinical Development Division, Kangstem Biotech Co., Ltd, 512 Teheran-ro, Gangnam-Gu, Seoul, 06179 Republic of Korea; 3 Department of Statistics, Graduate School, Sungkyunkwan University, 25-2, Seonggyungwan-Ro, Jongno-Gu, Seoul, 03063 Republic of Korea

**Keywords:** Long-term care, Potentially inappropriate medications, Older adults, Outpatient visit, Cohort study

## Abstract

**Background:**

With an increase in the aging population, the number of older adults who require long-term care (LTC) is growing, enhancing drug-related issues. The reduced capacity of LTC users to precisely utilize medical services poses additional challenges owing to restrictions in daily activities. We compared older adults who required LTC with those who did not require LTC to confirm differences in the use of potentially inappropriate medications (PIMs), frequently used PIMs, and associating factors in Korea.

**Methods:**

Using the Korean National Health Insurance Service cohort data, adults aged ≥ 65 years as of 2017 who were LTC beneficiaries (at home and LTC facilities) were selected and matched 1:1 with a control group (LTC non-beneficiaries). PIM was defined based on the 2019 American Society of Geriatrics Beers criteria. PIM use and medical resource utilization according to LTC requirements were compared for one year after the index date. After correcting for other confounding variables, differences in the risk of PIM use on person-based according to LTC eligibility were assessed using multivariate logistic regression.

**Results:**

Among the 13,251 older adults requiring LTC in 2017, 9682 were matched with counterparts and included. Among those who received an outpatient prescription including PIM at least once yearly, 83.6 and 87.6% were LTC beneficiaries and LTC non-beneficiaries, respectively (*p* < 0.001). Using the number of outpatient prescriptions as the baseline, 37.2 and 33.2% were LTC beneficiaries and LTC non-beneficiaries, respectively (*p* < 0.001). In both groups, elevated PIM use depended on increased medical resource utilization, as shown by increased outpatient visits and medical care institutions visited.

Adjusting other influencing factors, the need for LTC did not significantly associated with PIM use (odds ratio [OR] 0.93, 95% confidence interval [CI] 0.84–1.04); the number of drugs consumed (3–4: OR 1.42, 95% CI 1.25–1.61; 5–9: OR 2.24, 95% CI 1.98–2.53; 10 and more: OR 3.72, 95% CI 3.03–4.55; reference group: 2 and less), frequency of visits (7–15: OR 1.95, 95% CI 1.71–2.23; 16–26: OR 3.51, 95% CI 3.02–4.07; 27–42: OR 5.84, 95% CI 4.84–7.05; 43 and more: OR 10.30, 95% CI 8.15–13.01; reference group: 6 and less), and visits to multiple medical care institutions (3–4: OR 1.96, 95% CI 1.76–2.19; 5 and more: OR 3.21, 95% CI 2.76–3.73; reference group: 2 and less) emerged as primary influencing factors. PIMs mainly prescribed included first-generation antihistamines, benzodiazepines, and Z-drugs in both groups; quetiapine ranked second-highest among LTC beneficiaries.

**Conclusions:**

The LTC demand did not significantly associated with PIM utilization. However, the number of drugs consumed, and the pattern of medical resource use were important factors, regardless of LTC requirements. This highlights the need to implement comprehensive drug management focusing on patients receiving polypharmacy and visiting multiple care institutions, regardless of LTC needs.

## Background

With an increase in the aging population, the number of older adults requiring long-term care (LTC) has also significantly increased and is projected to rise by 100 million worldwide by 2030 compared with that in 2015 [1]. Accordingly, Germany, Japan, Korea, and other countries have implemented LTC services for their older populations as part of national healthcare. In Korea, LTC insurance (LTCI) was introduced in 2008 and operated by the National Health Insurance Service (NHIS). The main factors for adopting LTCI were rapid aging and the high healthcare expenditure for older adults due to their longevity and chronic disease [2]. Beneficiaries of the LTCI primarily target older adults (≥ 65) and the younger population requiring LTC. NHIS also operates the care need certification (CNC) system, a standardized 52-item functional assessment tool and procedure, to assess the applicant's eligibility [3]. It is a six-level system from level 1 (totally dependent) to 4 (moderately dependent), followed by levels 5 (dementia with mild dependency) and 6 (cognition assistance) based on the CNC rating score [2, 4]; LTC service benefits differ based on the level. As of 2020, there were 860,000 beneficiaries, corresponding to 10.1% of older population, and this number is growing annually [4].

Older adults often experience multimorbidity [5], possibly presenting several drug-related problems, such as polypharmacy or potentially inappropriate medications (PIMs). PIMs increase the risk of fractures, hospitalization, and death [6]; this is an important factor for the awareness of inappropriate drug use among older adults. PIM exposure was reported in 43% of older adults living in LTC facilities [7], which is more than 20% higher than noted among older adults living in the local community [8, 9]; this indicated a much higher risk of PIM use among older adults living in facilities. The main factors influencing PIM use among older adults living in nursing facilities include polypharmacy, concomitant diseases, such as falls, fractures, and chronic diseases [10–13]. Moreover, although the risk of PIM use is known to increase in patients with dementia or other mental illnesses that require care [14, 15], one study has reported that the risk of PIM use decreases following nursing home admission in patients with dementia [16]; however, this conclusion remains controversial.

Considering older adults in need of LTC, access to medical care remains challenging, given the restrictions in their daily living activities. Older adults who need LTC have a higher prevalence of chronic diseases than those without LTC needs [17], along with poor access to medical care, such as visiting specialists [18, 19]. According to previous studies, while the number of outpatient visits among older adults LTC beneficiary was lower than that among LTC non-beneficiaries (28.8 *vs.* 32.8, *p* < 0.001), the number of days in the hospital (70.0 *vs.* 48.9, *p* < 0.001), number of medications taken (4.7 *vs*. 3.8, *p* < 0.001), and number of days of prescription (280.9 *vs*. 277.1, *p* < 0.001) was higher than that among LTC non-beneficiaries [20]. Medical care was found to be associated with PIM use. The risk of PIM use increases with high medical care utilization [21] and low continuity of care [22, 23]. However, only a few studies have compared PIM use in older adults requiring LTC and those who do not warrant LTC. Furthermore, no previous report has comparatively analyzed PIM use considering patterns in medical care use.

The objective of the present study was to determine whether the PIM utilization differed between older adults with LTC needs and those without such needs, identify influencing factors, and confirm differences in frequently used PIM ingredients. Furthermore, whether the medical utilization patterns differed based on the need for LTC and whether they were associated with PIM utilization were examined.

## Methods

### Data

We used the National Health Insurance Service (NHIS-NSC (2002–2015) sample cohort data for this matched cohort study. Data were obtained by undertaking the stratified sampling of approximately 2% of the national population eligible for health insurance and medical aid beneficiaries as of 2006, based on gender, age, insurance type, insurance premium decile, and region; this was subsequently constructed as research data as a cohort indicating socioeconomic status, medical care usage, medical institution visits, and LTC insurance status of sampled subjects (approximately 1 million) from 2002 to 2019 [24]. Individuals within the cohort were anonymized. The data included eligibility data such as gender, age, and insurance premium decile of each subject, as well as medical care usages, such as hospitalization, outpatient treatment, drug prescription, information of LTCI such as the rating score based on the CNC system and assigned level for LTC service benefit and history of LTC service use.

### Study populations

We defined older adults based on LTC needs as LTC beneficiaries and non-LTC beneficiaries as counterparts that did not require LTC. Older adults who met LTCI eligibility and were assigned with LTCI levels were defined as LTC beneficiaries. We evaluated the overall effects of LTC needs without distinguishing between facility and at-home LTC beneficiaries. The LTC level of beneficiaries was defined as the index date level. Participants were selected in 2017, the most recent year before the LTCI levels changed in 2018 (from levels 5 to 6).

**Fig. 1:**
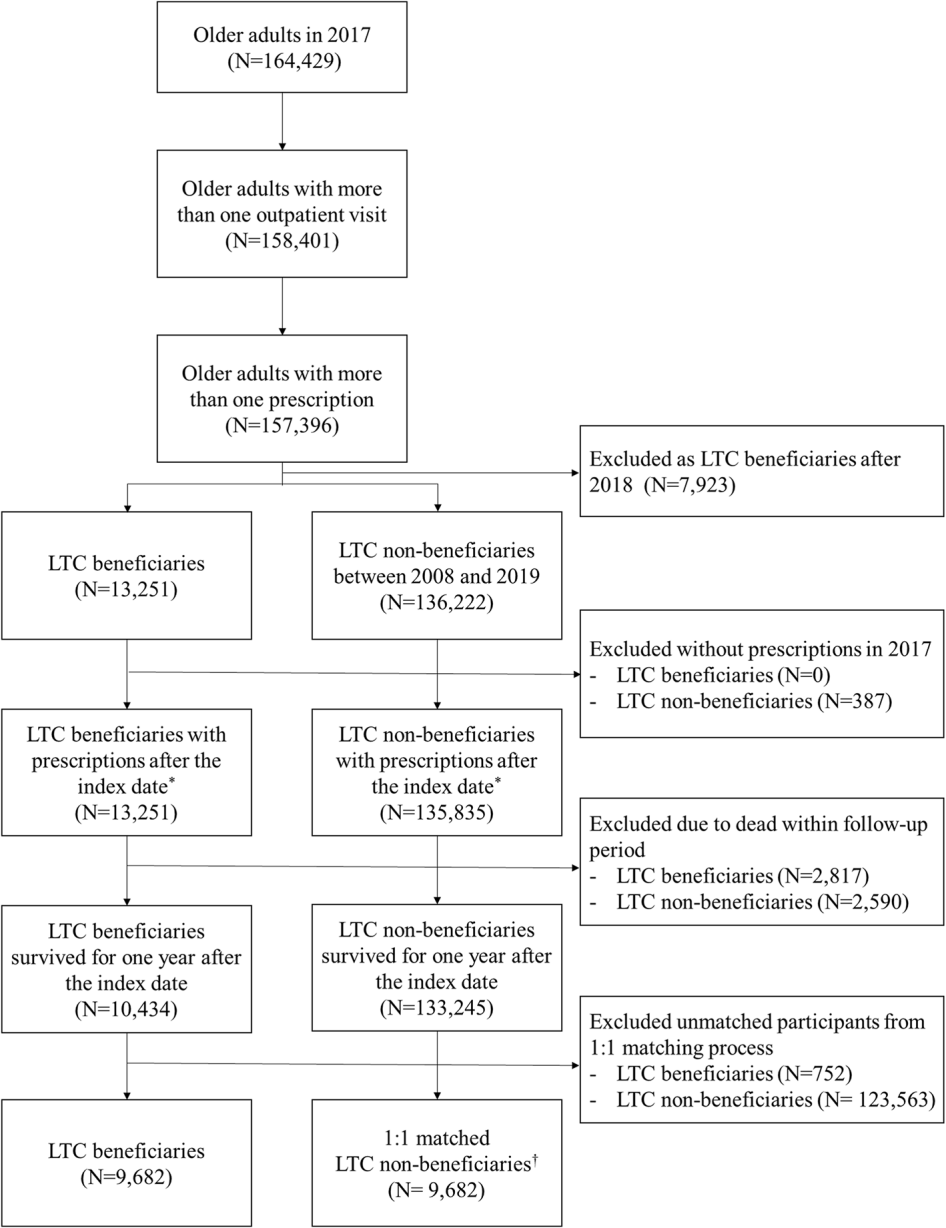
Flowchart of the study population: long-term care beneficiaries and matched counterparts. LTC: Long-term care. * Index date: first date of prescription in 2017. † LTC non-beneficiaries were 1:1 matched to LTC beneficiaries by sex, age, type of insurance, and Charlson comorbidity index

In the present study, older adults with outpatient visits and who received prescriptions were selected from 164,429 older adults (aged 65–100 years) as of 2017. Among them, LTC beneficiaries were defined as the target group (*n* = 13,251), and non-LTC beneficiaries, that is, older adults who had never received an LTCI service from 2008 to 2019, were used as the control group (*n* = 136,222). Although older adults were participants requiring LTC in 2017, time might be required to evaluate and accredit LTC beneficiaries. We used LTC non-beneficiaries until 2019 as the control group to minimize this effect (Fig. 1). The index date was set as the first day of the drug prescription in 2017. Subjects who had never received an outpatient prescription in 2017 or died within one year after the index date were excluded from the analysis. LTC and non-LTC beneficiaries were matched 1:1 using a propensity score. Age, sex, Charlson comorbidity index (CCI), and health insurance type were used for matching.

### Potentially inappropriate medications, PIMs

We defined PIMs based on the 2019 American Society of Geriatrics (AGS) Beers criteria [25]. The Beers criteria were first developed in 1991 in the United States and, since 2011, have been updated every three years. It is the most widely used explicit criterion as a guideline for drugs that should be avoided in the older population or patients with certain diseases. However, among drugs subject to the Beers criteria, some are either not used or are exclusively used in Korea. Therefore, medicines for older adults (as of July 2021) provided by the Korea Institute of Drug Safety and Risk Management were also considered PIMs. Considering additional recommendations for some drugs using the 2019 Beers criteria, PIM is defined as follows: Drugs are considered PIMs if they are related to peripheral alpha-1 blockers and central alpha-blockers for use in patients with hypertension (ICD 10: I10–I13, I15). Dronedarone and digoxin are defined as PIMs if used in patients with atrial fibrillation (I48) and heart failure (I11.0, I13.0, I13.2, I50.x). Amiodarone is deemed as a PIM when employed in patients with atrial fibrillation (I48). Antipsychotics are considered PIMs when used in patients with dementia or when the disease code is not Fxx (mental and behavioral disorders). Proton-pump inhibitors are defined as PIMs when used for more than 8 weeks (continuous prescription within a 15-day gap) and excluded when used concurrently with oral corticosteroids or nonsteroidal anti-inflammatory drugs (NSAIDs). Chronic NSAID use was defined as a PIM when used for more than 60 days (continuous prescription within a 15-day gap). Outpatient prescriptions were targeted, and only oral drugs and injections, excluding medicine for external use, such as creams, ointments, and ophthalmic drugs, were analyzed. Medications taken during hospitalization are likely for treating acute and severe diseases and may be based on a more professional medical decision. Therefore, we included only outpatient prescriptions in the analysis.

The prevalence of PIM based on person was defined as the proportion of patients (numerator) who received prescriptions for PIMs at least once during the year after the index date among those who had outpatient drug prescriptions (denominator). Based on the prescription, it was defined as the prescription (numerator) containing the PIM among all outpatient prescriptions (denominator) received by the patient during the year after the index date. The top 10 most prescribed PIMs were identified based on prescriptions for LTC beneficiaries and matched controls.

### Medical care utilization and prescription

The use of medical care was classified into inpatient and outpatient visits, hospitalization at least once yearly, and the number of outpatient visits. Based on the classification by quantiles in previous studies [26, 27], the number of outpatient visits was divided into five sections using the quintile of the number of outpatient visits for LTC beneficiaries: 6 times or less, 7–15 times, 16–26 times, 27–42 times, and 43 times or more. The number of medical institutions visited referred to the number of medical centers visited for one year per person and was divided into three groups: ≤ 2, 3–4, and ≥ 5. The predominant medical center was defined as the type of medical institution that was most visited during the year.

The number of drugs consumed was calculated based on the outpatient prescriptions. The total number of prescription days per year was calculated using the longest number of prescription days for each prescription. The number of drugs consumed was calculated by summing the number of prescription days for each drug and then dividing it by the total number of prescription days (up to 365 days). Taking five and more drugs usually defined as polypharmacy and ten and more drugs defined as excessive polypharmacy [28], the number of drugs consumed was divided into four groups: ≤ 2, 3–4, 5–9, and ≥ 10.

### Covariates

Patient age was classified into four groups: 65–74 years, 75–84 years, 85–89 years, and 90 years and older. Insurance types were classified as NHI and medical aid. Medical aid is a medical assistance program for low-income individuals. The CCI was calculated based on the main diagnosis code of claim data for one year prior to the index date with two or more hospitalizations and two or more outpatient visits. The CCI was subsequently classified into six groups: 0, 1, 2, 3, 4, and 5 + scores. In general, three and more CCI indicates a higher risk of adverse health outcomes [29, 30]; however, we further divided it into six groups to similarly match the risk levels. Chronic conditions (e.g., hypertension, diabetes) were determined by defining the disease as having two or more hospitalizations and two or more outpatient visits based on the main diagnosis and sub-diagnosis in the year prior to the index date. Dementia was defined as the presence of dementia drugs (memantine, rivastigmine, galantamine, and donepezil), given that the diagnostic code of mental and behavioral disorders was masked as Fxx in the cohort data. For the diagnosis code, the Korean standard disease/sign code 7 was used, according to the following disease codes: cerebrovascular disease (I60–I69), hypertension (I10–I13, I15), diabetes (E10–E14), hyperlipidemia (E78), Parkinson’s disease (G20–G23), cardiovascular disease (I05–I09, I20–I27, I30–I52), and osteoarthritis (M00–M19, M45).

### Statistical analysis

We performed a descriptive analysis of basic characteristics and medical care utilization. For comparison between LTC beneficiaries and LTC non-beneficiaries, the chi-square test was used for categorical data, and the t-test or ANOVA was used for continuous data. Logistic regression analysis was applied to analyze the association between the receipt of LTC services and PIM use on a person-based. Statistical significance was set at a 5% significance, and 95% confidence intervals (CIs) were used. All analyses were performed using SAS Enterprise 7.1 (SAS Inc., Cary, NC).

## Results

### Characteristics of participants

Table 1 presents the characteristics of matched 9682 LTC beneficiaries among 13,251 LTC beneficiaries in 2017. Among LTC beneficiaries, 73.9% were female, and those aged 75–84 years accounted for the largest proportion (49.2%). Medical aid recipients accounted for 14.9% of the total, exceeding the national average of 3%. A CCI score of 1 was most commonly observed (35.0%); among chronic diseases, hypertension was the most frequently noted (53.7%), followed by dementia in 45.7% of all beneficiaries. Most patients had level 4 LTC (40.4%).

**Table 1:** Characteristics of LTC beneficiaries and LTC non-beneficiaries

Variables		LTC beneficiaries		LTC non-beneficiaries		*p*-value
		n	%	n	%	
Number of patients		9682		9682		
Gender	Male	2527	26.1	2537	26.2	0.8701
	Female	7155	73.9	7145	73.8	
Age	65–74	1376	14.2	1376	14.2	0.9996
	75–84	4759	49.2	4752	49.1	
	85–89	2420	25.0	2424	25.0	
	≥ 90	1127	11.6	1130	11.7	
Type of health insurance	Health insurance	8236	85.1	8241	85.1	0.9196
	Medical aid	1446	14.9	1441	14.9	
CCI	0	1922	19.9	1921	19.8	0.9999
	1	3385	35.0	3381	34.9	
	2	2285	23.6	2283	23.6	
	3	1141	11.8	1150	11.9	
	4	577	6.0	572	5.9	
	≥ 5	372	3.8	375	3.9	
Chronic conditions	Hypertension	5194	53.7	5776	59.7	< .0001
	Dementia	4427	45.7	705	7.3	< .0001
	Osteoarthritis	2992	30.9	3958	40.9	< .0001
	Diabetes	2498	25.8	2980	30.8	< .0001
	Cerebrovascular disease	2497	25.8	1251	12.9	< .0001
	Cardiovascular disease	1679	17.3	1737	17.9	0.2742
	Hyperlipidemia	1570	8.1	2241	23.2	< .0001
	Mental/behavioral disorders^*^	931	9.6	921	9.5	0.807
	Parkinson’s disease	788	8.1	100	1.0	< .0001
LTC level	1	456	4.7	-		
	2	1097	11.3	-		
	3	3397	35.1	-		
	4	3913	40.4	-		
	5	819	8.5	-		

*CCI* Charlson comorbidity index, *LTC* long-term care

^*^ Mental/behavioral disorders excluding dementia and Parkinson’s disease

### Medical care utilization patterns

Table 2 presents patterns of medical care utilization and the number of drugs consumed. The incidence of hospitalization was significantly higher among LTC beneficiaries than among LTC non-beneficiaries (44.2 and 27.5% for LTC beneficiaries and LTC non-beneficiaries, respectively; *p* < 0.001). The number of outpatient visits was 32.3 (standard deviation [SD] 34.0) for LTC beneficiaries and 33.4 (SD 33.4) for LTC non-beneficiaries. The number of medical institutions visited was significantly higher among LTC non-beneficiaries than among LTC beneficiaries (4.2 and 5.4 for LTC beneficiaries and LTC non-beneficiaries, respectively; *p* < 0.001). Considering the predominant medical center type visited, 25.9% of beneficiaries visited secondary hospitals or higher, whereas only 11.9% of LTC non-beneficiaries visited such institutions. Overall, 62.9 and 46.8% of LTC beneficiaries and LTC non-beneficiaries were consuming five or more drugs, respectively. Furthermore, 21.4 and 10.8% of LTC beneficiaries and LTC non-beneficiaries were consuming 10 or more drugs.

**Table 2:** Medical care utilization among LTC beneficiaries and LTC non-beneficiaries

		LTC beneficiaries		LTC non-beneficiaries		*p*-value
		n	%	n	%	
Hospitalization	Yes	4276	44.2	2664	27.5	< 0001
	No	5406	55.8	7018	72.5	
Number of outpatient visits	mean (SD)	32.3 (34.0)		38.0 (33.4)		< 0001
	≤ 6	1023	10.6	533	5.5	< 0001
	7–15	2176	22.5	1626	16.8	
	16–26	2679	27.7	2244	23.2	
	27–42	1896	19.6	2330	24.1	
	≥ 43	1908	19.7	2949	30.5	
Number of medical institutions visited	mean (SD)	4.2 (2.8)		5.4 (3.1)		< 0001
	≤ 2	3082	31.8	1714	17.7	< 0001
	3–4	3166	32.7	2652	27.4	
	≥ 5	3434	35.5	5316	54.9	
Type of predominant medical center	Tertiary hospital	778	5.9	436	3.8	< 0001
	Secondary hospital	1938	20.0	785	8.1	
	Hospital	929	9.6	348	3.6	
	Clinic	6037	62.4	8113	83.8	
Number of drugs consumed	≤ 2	1979	20.4	3051	31.5	< 0001
	3–4	1620	16.7	2103	21.7	
	5–9	4015	41.5	3485	36.0	
	≥ 10	2068	21.4	1043	10.8	

*LTC* long-term care, *SD* standard deviation

### Prevalence of PIMs

The annual prevalence of PIMs was 83.6% (95% CI 81.8–85.5%) and 87.6% (95% CI 85.7–89.4%) among LTC beneficiaries and LTC non-beneficiaries, respectively (Table 3). Based on the prescription measures, PIM prescriptions accounted for 37.2% (95% CI 37.0–37.4%) among beneficiaries and 33.2% (95% CI 33.0–33.4%) among LTC non-beneficiaries. No significant differences in the average number of PIM prescriptions per person were detected between LTC beneficiaries and LTC non-beneficiaries (13.1 and 13.5, respectively; *p* = 0.054).

**Table 3:** Prevalence of PIM among LTC beneficiaries and LTC non-beneficiaries

		LTC beneficiaries (*N* = 9682)		LTC non-beneficiaries (*N* = 9682)		*p*-value
		N	%	N	%	
PIM use (person-based)		8097	83.6	8479	87.6	< 0001
PIM use (prescription-based)		105,719/284,045	37.2	114,202/343,848	33.2	< 0001
PIM prescriptions per person,mean (SD)		13.1	(13.4)	13.5	(14.1)	0.0537
Number of PIM prescriptions (person-based)	1–2	1246	15.39	1245	14.68	< 0001
	3–5	1254	15.49	1460	17.22	
	6–13	2763	34.12	2731	32.21	
	14–19	1251	15.45	1194	14.08	
	≥ 20	1583	19.55	1849	21.81	

*LTC* long-term care, *PIM* potentially inappropriate medications, *SD* standard deviation

The use of PIMs in both groups exhibited an increasing pattern as the number of outpatient visits and visits to medical institutions increased (Table 4). In the LTC beneficiary group, the prevalence of PIM was 52.9 and 96.5% when outpatient visits were ≤ 6 and ≥ 43, respectively (43.5 and 98.0% in the LTC non-beneficiary group, respectively). The prevalence of PIM in the LTC beneficiary group was 68.6% when the number of visited medical institutions was less than two; however, this value increased to 94.6% when more than five medical institutions were visited (64.1 and 95.8% of the LTC non-beneficiary group, respectively).

**Table 4:** Prevalence of PIM according to medical care utilization

		LTC beneficiaries			LTC non-beneficiaries			*p*-value
		Total (N)	PIM user (N)	%	Total (N)	PIM user (N)	%	
Number of outpatient visits	≤ 6	1023	541	52.9	533	232	43.5	< 0.001
	7–15	2176	1591	73.1	1626	1181	72.6	0.740
	16–26	2679	2346	87.6	2244	1970	87.8	0.815
	27–42	1896	1777	93.7	2330	2205	94.6	0.206
	≥ 43	1908	1842	96.5	2949	2891	98.0	0.001
Number of medical institutions visited	≤ 2	3082	2113	68.6	1714	1098	64.1	0.002
	3–4	3166	2734	86.4	2652	2289	86.3	0.962
	≥ 5	3434	3250	94.6	5316	5092	95.8	0.013

*LTC* long-term care, *PIM* potentially inappropriate medications

In both groups, ingredients corresponding to the most commonly used PIMs were first-generation antihistamines, benzodiazepines, and Z-drugs (Table 5). However, in the case of LTC beneficiaries, the use of quetiapine, an atypical antipsychotic, was ranked second-highest.

**Table 5:** Top 10 PIMs

Ranking	LTC beneficiaries (N^*^ = 105,719)			LTC Non-beneficiaries (N^*^ = 114,202)		
	Name of ingredients	N^*^	%	Name of ingredients	N^*^	%
1	Chlorpheniramine	24,768	23.43	Chlorpheniramine	39,130	34.26
2	Quetiapine	8427	7.97	Diazepam	10,746	9.41
3	Diazepam	7414	7.01	Glimepiride	8722	7.64
4	Zolpidem	5997	5.67	Dimenhydrinate	5201	4.55
5	Glimepiride	5937	5.62	Hydroxyzine	4890	4.28
6	Amitriptyline	5108	4.83	Zolpidem	4633	4.06
7	Hydroxyzine	4263	4.03	Celecoxib	3847	3.37
8	Dimenhydrinate	4077	3.86	Piprinhydrinate	3741	3.28
9	Aceclofenac	2493	2.36	Amitriptyline	3536	3.1
10	Solifenacin	2398	2.27	Aceclofenac	3367	2.95

*LTC* long-term care, *PIM* potentially inappropriate medications

^*^Number of PIM prescriptions in each group

### Factors associated with PIM use

Logistic regression analysis was performed to identify factors influencing PIM use (Table 6). On adjusting other confounders, LTC did not significantly associate with the likelihood of PIM use (OR 0.93, 95% CI 0.84–1.04). Notably, factors related to medical care utilization were a major associated factor for PIM use. The likelihood of PIM use increased with an increasing number of outpatient visits (7–15 times: OR 1.95, 95% CI 1.71–2.23; 16–26 times: OR 3.51, 95% CI 3.02–4.07; 27–42 times: OR 5.84, 95% CI 4.84–7.05; more than 43 times: OR 10.30, 95% CI 8.15–13.01; reference group: less than 6 times) and an increasing number of medical institutions visited (3–4 institutions: OR 1.96, 95% CI 1.76–2.19; more than five institutions: OR 3.21, 95% CI 2.76–3.73; reference group: less than 2 institutions). Considering the predominant medical center visited, the likelihood of PIM use increased on visiting medical centers other than tertiary hospitals. As the number of drugs consumed increased, the risk of PIM use also increased. Among chronic conditions, increased risk of PIM use was observed in the presence of mental/behavioral disorders (OR 1.43, 95% CI 1.16–1.75) and osteoarthritis (OR 1.48, 95% CI 1.32–1.67).

**Table 6:** Factors associated with PIM use

		adjusted OR	95% CI		*p*-value
LTC beneficiaries	No	1			
	Yes	0.93	0.84	1.04	0.1855
Hospitalization	Yes	1			
	No	1.08	0.97	1.19	0.1564
Number of outpatient visits	≤ 6	1			
	7–15	1.95	1.71	2.23	< 0.0001
	16–26	3.51	3.02	4.07	< 0.0001
	27–42	5.84	4.84	7.05	< 0.0001
	≥ 43	10.30	8.15	13.01	< 0.0001
Type of predominant medical center	Tertiary hospital	1			
	Secondary hospital	1.36	1.15	1.60	0.0004
	Hospital	1.84	1.50	2.26	< 0.0001
	Clinic	1.51	1.29	1.77	< 0.0001
Number of medical institutions visited	≤ 2	1			
	3–4	1.96	1.76	2.19	< 0.0001
	≥ 5	3.21	2.76	3.73	< 0.0001
Number of drugs consumed	≤ 2	1			
	3–4	1.42	1.25	1.61	< 0.0001
	5–9	2.24	1.98	2.53	< 0.0001
	≥ 10	3.72	3.03	4.55	< 0.0001
Chronic conditions (Reference: No)	Dementia	1.09	0.97	1.22	0.1532
	Mental/behavioral disorders^*^	1.43	1.16	1.75	0.0008
	Cerebrovascular disease	0.75	0.66	0.86	< 0.0001
	Cardiovascular disease	0.63	0.54	0.73	< 0.0001
	Parkinson’s disease	0.91	0.71	1.19	0.5074
	Hypertension	0.85	0.77	0.93	0.0005
	Diabetes	1.13	1.00	1.27	0.0414
	Hyperlipidemia	0.86	0.76	0.97	0.0166
	Osteoarthritis	1.48	1.32	1.67	< 0.0001

*CI* confidence interval, *LTC* long-term care, *OR* odds ratio, *PIM* potentially inappropriate medications

^†^LTC non-beneficiaries were matched for each case by sex, age, CCI score, and insurance type. Therefore, we excluded the variables used for matching from the adjusted OR estimation

^*^ Mental/behavioral disorders excluding dementia and Parkinson’s disease

## Discussion

Herein, we compared participants requiring LTC (LTC beneficiaries) and those without LTC requirements (non-LTC beneficiaries) to evaluate the effects of LTC need on medical care and PIM use. LTC beneficiaries experienced a greater number of hospitalizations (44.2% *vs*. 27.5%, *p* < 0.001) and fewer outpatient visits (32.3 *vs*. 38.0, *p* < 0.001) than non-LTC beneficiaries. The number of outpatient visits was relatively small (4.2 *vs*. 5.4, *p* < 0.001), with more visits to a secondary hospital level or higher considering the predominant medical center visited (25.9% *vs*. 11.9%, *p* < 0.001). These results are similar to those of previous studies, showing that medical care utilization was low among older adults with LTC needs (Schulz et al.). In contrast to the results of Schulz et al. indicating that medical care use is limited owing to relatively few specialist visits, our results revealed no reduction in access to high-level medical care institutions. A characteristic of the health care system in Korea is the absence of a primary care system, and outpatient visits to secondary/tertiary medical institutions are readily available according to the patient/guardian’s choice. In addition, in Korea, patients who experience difficulty in mobility can receive outpatient prescriptions through a guardian’s surrogate visit, likely leading to continuous prescriptions from high-level hospitals.

The prevalence of PIMs among older adults LTC beneficiaries was 83.6%, which was lower than that among matched counterparts (87.6%) (*p* < 0.001). However, based on prescriptions, the prevalence of PIMs was 37.2 and 33.2% in LTC beneficiaries and LTC non-beneficiaries, respectively, indicating a higher prevalence among LTC beneficiaries than among LTC non-beneficiaries (*p* < 0.001). No marginally significant difference in PIM prescriptions per person was observed between LTC beneficiaries and non-beneficiaries (13.1 and 13.5, *p* < 0.054). Besides, the number of total prescriptions in LTC beneficiaries was relatively small; thus, the prescription-based prevalence of PIMs was higher in LTC beneficiaries than non-beneficiaries. Following logistic analysis after adjusting for other factors, we detected no significant difference between the two groups considering the likelihood of PIM use (OR 0.93, 95% CI 0.84, 1.04). A previous study compared older adults housed in residential aged care facilities (RACF) with those in the local community and found no significant difference in more than one PIM prescription (*p* = 0.09); however, the average number of PIMs was significantly higher in older adults living in RACFs than among those in the local community (1.96 *vs*. 1.26, *p* < 0.05) [10].

Conversely, regardless of LTC, the likelihood of PIM increased with polypharmacy, increasing the number of outpatient visits and visits to various medical institutions. Polypharmacy is considered a key factor in PIM use, and our results were consistent with those of previous studies [31, 32]. One possible explanation for the high prevalence of PIM utilization was the high share of taking > 5 medications among older adults: 70.2% in Korea, whereas the OECD average was 46.7% [33]. In line with the OECD report, LTC beneficiaries with polypharmacy (≥ 5 drugs) accounted for 62.9% of this study. Polypharmacy was partially explained by the cultural preference for taking medications [34, 35]. Fragmented Korea’s medical system without a general practitioner acting as a gatekeeper could lead to polypharmacy and redundant use of medical care. In an analysis conducted in Taiwan, an increasing number of medical care visits and doctors visited was reported as a significant factor that increased the likelihood of PIM use (OR 1.31, 95% CI 1.18–1.46; OR 1.15, 95% CI 1.03–1.28, respectively) [21]. In addition, PIM use increased when the patient visited multiple medical care institutions (OR 1.64, 95% CI 1.50–1.79) [21], along with poor continuity of care. These characteristics have been noted in countries lacking primary-care gatekeeper system [22, 36]. However, in the group with low medical utilization and few medical institutions visited, PIM use was relatively high among LTC beneficiaries in this study; therefore, further in-depth analysis is required.

First-generation antihistamines, benzodiazepines, and Z-drugs were the most commonly prescribed PIMs. Our results were consistent with those of previous studies. Psychotropic drugs and first‐generation antihistamines were the most commonly used PIMs in Taiwanese older adults [21]. Antihistamines accounted for the highly used PIMs in Asian countries [37, 38]. Benzodiazepines were frequently used in European countries [39]. In Korea, first‐generation antihistamines were likely prescribed for common colds [40]. Jang et al. explained that the cheaper price of first‐ than second- and third-generation antihistamines could result in their frequent prescriptions along with the Korean healthcare system, where it is easy to visit medical institutions [22]. However, the use of an atypical antipsychotic drug, quetiapine, was more common among older adults requiring LTC (Top 2 in PIM). Quetiapine is used to treat schizophrenia and bipolar disorders [41, 42] and is employed off-label in older patients with dementia to control mental and behavioral symptoms, as well as for psychotic depression in Parkinson's disease [43]. However, the use of antipsychotics is known to increase mortality in patients with senile dementia [44, 45] and the risk of cerebrovascular adverse events [46, 47]. Similar to our study, the high-frequency ingredients based on the EU-PIM list were "proton-pump inhibitors used longer than 8 weeks,” followed by "risperidone used longer than 6 weeks,” thereby confirming the frequent use of antipsychotics for dementia [48]. Frequent prescription of antipsychotic and sedative PIMs among older adults needing LTC was associated with chronic conditions, such as dementia, cerebrovascular disease, and Parkinson's disease. Approximately 45% of LTC beneficiaries presented with dementia, and quetiapine usage was high; therefore, careful attention should be paid to the potential risk of adverse outcomes.

The drug utilization review (DUR) program for cautiously using medications for older adults was implemented in October 2015 in Korea, considering the population’s rapid aging and the vulnerability of older adults to medication harm. Relevant information is presented in a pop-up window, and physicians should select the reason for prescribing DUR-listed drugs. This prospective DUR program reduced the prescription of DUR-listed drugs by 0.49% (95% CI -0.60, -0.37); however, medications in the DUR program were small; thus, the overall effect was expected to be limited [49]. Several ongoing efforts exist to develop and introduce comprehensive medication reviews in Korea. The pilot project for polypharmacy management has been implemented [50], and a medication review tool and eligibility criteria for residents in LTC facilities have been recently developed [51, 52]. Furthermore, comprehensive medication management is necessary to consider the healthcare characteristics in Korea, where no primary-care gatekeeper system is available.

This study had several limitations. First, due to the characteristics of claims data, PIM underestimation is possibly caused by non-coverage and exclusion of over-the-counter drugs from the data. Second, the physician’s rationale for prescribing PIM is unknown, given the lack of clinical data. Accordingly, we could not distinguish cases in which PIMs were prescribed because the benefit outweighed the medication risk. However, we considered the disease or conditions mentioned in the Beers criteria for defining the PIM utilization to reflect the clinical status. Third, dementia was defined as the prescription of dementia drugs because the diagnostic code related to mental disorders was masked as Fxx in the data. However, these drugs were indicated only for dementia; therefore, the measurement error would be inconsequential. Fourth, no distinction was established between facility- and at-home LTC beneficiaries. However, according to Schulz and his colleagues, both facility- and at-home LTC users had limitations in medical use when compared with healthy older adults[19]; therefore, the patterns of the two groups were expected to be similar. Furthermore, by not distinguishing between facility and at-home LTC beneficiaries, their association with LTC needs can be assessed on their own. Finally, we did not confirm whether PIM utilization impacted adverse health outcomes. Accordingly, further follow-up studies are required.

## Conclusion

PIM use among older adults was very high, either with or without LTC needs in Korea, and was associated with medical care utilization patterns, polypharmacy, and some diseases but with the LTC need. This finding suggests that PIM use in older adults requiring LTC should be reviewed from a multidimensional perspective.

## Data Availability

This study used the National Health Insurance Service – national sample cohort (NHIS-NSC) data (NHIS-2021–2-249). These third-party data were obtained from the Korean National Health Insurance Service (KNHIS). The authors had no special access privileges to the data. Interested, qualified researchers can apply for access to the data by contacting the KNHIS (https://nhiss.nhis.or.kr/bd/ab/bdaba001cv.do).

## Abbreviations

AGS: American Society of Geriatrics
CCI: Charlson comorbidity index
CIs: Confidence intervals
CNC: Care need certification
DUR: Drug utilization review
LTC: Long-term care
LTCF: Long-term care facility
LTCI: Long-term care insurance
NHIS: National Health Insurance Service
NSAIDs: Nonsteroidal anti-inflammatory drugs
OR: Odds ratio
PIMs: Potentially inappropriate medications
RACF: Residential aged care facilities
SD: Standard deviation
